# Antimicrobial and Antioxidant Activity of Chitosan/Hydroxypropyl Methylcellulose Film-Forming Hydrosols Hydrolyzed by Cellulase

**DOI:** 10.3390/ijms17091436

**Published:** 2016-09-06

**Authors:** Anna Zimoch-Korzycka, Łukasz Bobak, Andrzej Jarmoluk

**Affiliations:** Department of Animal Products Technology and Quality Management, Wrocław University of Environmental and Life Sciences, 37 Chelmonskiego St., 51-630 Wrocław, Poland; lukasz.bobak@up.wroc.pl (Ł.B.); andrzej.jarmoluk@up.wroc.pl (A.J.)

**Keywords:** chitosan, hydroxypropyl methylcellulose, cellulase, antioxidant properties, antimicrobial properties

## Abstract

The aim of this study was to evaluate the impact of cellulase (C) on the biological activity of chitosan/hydroxypropyl methylcellulose (CH/HPMC) film-forming hydrosols. The hydrolytic activity of cellulase in two concentrations (0.05% and 0.1%) was verified by determination of the progress of polysaccharide hydrolysis, based on viscosity measurement and reducing sugar-ends assay. The 2,2-diphenyl-1-picrylhydrazyl (DPPH) free radical scavenging effect, the ferric reducing antioxidant power (FRAP), and microbial reduction of *Pseudomonas fluorescens*, *Yersinia enterocolitica*, *Bacillus cereus*, and *Staphylococcus aureus* were studied. During the first 3 h of reaction, relative reducing sugar concentration increased progressively, and viscosity decreased rapidly. With increasing amount of enzyme from 0.05% to 0.1%, the reducing sugar concentration increased, and the viscosity decreased significantly. The scavenging effect of film-forming solutions was improved from 7.6% at time 0 and without enzyme to 52.1% for 0.1% cellulase after 20 h of reaction. A significant effect of cellulase addition and reaction time on antioxidant power of the tested film-forming solutions was also reported. Film-forming hydrosols with cellulase exhibited a bacteriostatic effect on all tested bacteria, causing a total reduction.

## 1. Introduction

Food safety is a priority for food production. However, consumers consider synthetic food additives as potential hazards and avoid them, which significantly limits the choice of preservatives. Therefore, food manufacturers are constantly looking for alternative sources of preservatives in the form of natural substances able to prolong shelf life and ensure a high level of food safety and consumer health [1]. The use of active packaging may solve the problem. Thus, the preparation of well-defined materials with antimicrobial activity or anti-biofouling ability will reduce the risk of contagion, contamination, or other threats [2].

Active packaging may change conditions within the package to extend the shelf life of the product. There are two types of these packages: absorbers and emitters. Absorbers include in their composition substances which eliminate the negative active material from the atmosphere. Oxygen, ethylene, carbon dioxide, moisture, and odors are the most absorbed substances. Emitters secrete substances that interact positively (bactericidal property). Carbon dioxide, antioxidants, and preservatives are emitted [3]. Both types of packaging are especially effective in the preservation of raw food products, such as fruits, vegetables, or meat.

Meat and meat products are ideal for the growth of bacteria, yeasts, and fungi. The microorganisms present in these products will reduce their quality, durability, and nutritional value [4]. Lactic acid bacteria, *Pseudomonas*, *Enterobacteriaceae*, *Escherichia*, *Salmonella*, *Listeria*, and *Yersinia* are responsible for decreasing the quality of meat and meat products. Microbial growth in stored meat often contributes to its undesirable organoleptic changes. For this reason, the meat industry is in great need to use packaging to prevent bacterial growth. The use of antimicrobial packaging can inhibit and prevent the growth of microorganisms during storage, thereby increasing the safety of meat products [5].

Chitosan inhibits the growth of bacteria, fungi, and yeasts [6], but exhibits the strongest activity against bacteria [7]. Chitosan has antimicrobial properties only in acidic solutions, because of its poor solubility in solutions of pH above 6.0 [8]. The polymer solubility and antimicrobial properties are enhanced in pH below 6.0, because the –NH_2_ at the C-2 position of glucosamine residues is positively charged [9]. There are a few hypotheses regarding chitosan′s antimicrobial mechanism of action. The first theory is associated with the interaction between the positive charge at the C-2 position of glucosamine chitosan and the negatively-charged cell wall of microorganisms [9,10]. Another hypothesis is associated with the incorporation of chitosan to the DNA molecule, which causes inhibition of mRNA synthesis [11]. The third mechanism is related to the chelation of metals which play a crucial role in stabilizing the cell wall [9]. The mode of action of high molecular weight chitosan and large particles relies on the interaction with the cell surface (resulting in changes in cell permeability [12]), or forming an impermeable layer around the cell (which blocks the transport of essential substances into the cell [13]). Many authors have proven better biological activity of chitooligomers than chitosan [14,15,16].

Cellulase is an endoglycosidase (EC 3.2.1.4.) that enables the hydrolysis of β-1,4 glucosidic bonds in polysaccharide chains—mainly cellulose. In view of its ability to cut the chain of chitosan or chitin, it is similar to chitinase [17]. Cellulase is widely produced in nature by many microorganisms and plants [18]. Cellulase produced from *Trichoderma reesei* is of greatest use in industry. There is also cellulase from *Aspergillus niger*, which is well characterized [19]. Microbial cellulases are used in food (for the extraction and clarification of fruit and vegetable juices to increase the yield of juices) and the feed industry (improving the digestibility of animal feeds), brewing (reduction in the degree of polymerization and wort viscosity), agriculture (for enhancing the growth of crops and controlling plant diseases), as well as in the pulp and paper industry, textile, laundry, and biofuel production [20].

The aim of the study was to evaluate the impact of cellulase on the biological activity of chitosan/hydroxypropyl methylcellulose film-forming hydrosols, by determining antioxidant and antimicrobial properties. The spectrum of bacteria was chosen from the most common food contaminants.

## 2. Results

### 2.1. Viscosity

**Figure 1:**
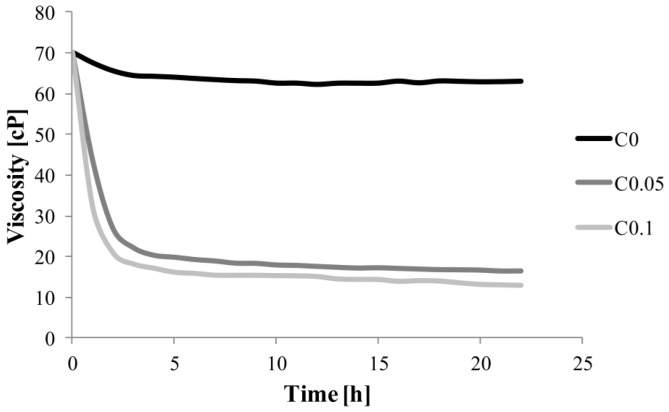
The effect of time (22 h) on viscosity of chitosan/hydroxypropyl methylcellulose hydrosols during enzymatic degradation by cellulase (C) in addition of 0%, 0.05% and 0.1% (C0, C0.05 and C0.1).

Monitoring chitosan/hydroxypropyl methylcellulose (CH/HPMC) blend hydrolysis was conducted by observing changes viscosity as a function of reaction time over the course of 22 h. The chosen hydrosol’s viscosity behavior is shown in Figure 1. It is observable that both enzyme concentrations (0.05% and 0.1%) displayed similar a hydrolysis reaction profile with polysaccharides, from 70 to 16 and 13 cP, respectively. The viscosity of the CH/HMPC hydrosol decreased sharply in the first 3 h of hydrolysis, followed by a relative constant value till the end of the 22 h reaction. An almost constant viscosity of the hydrosol without enzyme addition was observed (from 70 to 63 cP for 0 and 22 h, respectively). Il’ina et al. used enzyme preparation obtained from *Trichoderma reesei*, which possess β-glycanase, xylanase, and cellulase activities to hydrolyze 1% chitosan. After 24 h of reaction in 55 °C, the viscosity changed from 92 to 1.2 cP. They postulated that the reaction should be performed for 4 h in view of insignificant changes in viscosity values (1.3 cP) [21]. A probable explanation of such a kinetic reaction is depletion of the substrate. The hydroxypropyl methylcellulose is composed of glucose units linked by β-1,4 glycosidic bonds. The methyl, hydroxyl, and hydroxypropyl groups are distributed along the cellulose chain. The chitosan is composed of *N*-acetyl-d-glucosamine and d-glucosamine monomers linked by β-1,4 glycosidic bonds. The occurrence of the same bonds in the chains of HMPC and CH ensures the enzymatic action of cellulase. The β-1,4 glycosidic bonds of the chitosan molecule are cut off in the initial stage of hydrolysis, which is seen in the rapid viscosity decrease. Further reaction with the enzyme causes selective cross-degradation of the polysaccharide at a slow and stable rate [22]. Cellulase from *Trichoderma viridae* gave a 99% viscosity reduction after hydrolyzing carboxymethylcellulose (CMC) after 5 min at 50 °C. The higher reaction temperature caused rapid degradation of CMC [23]. In addition, cellulase from *T. ressei* against HPMC and chitosan at 24 °C reduced viscosity by about 70% in our study.

### 2.2. Reducing Sugar-Ends Content

**Figure 2:**
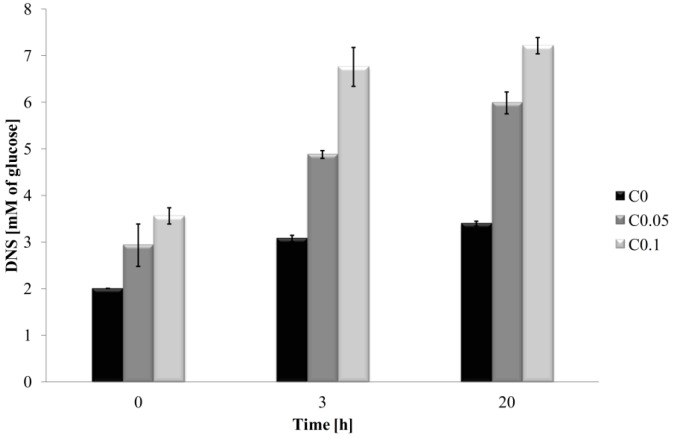
The effect of time on reducing sugar-ends during enzymatic degradation. DNS: 3,5-dinitrosalicylic acid.

Reducing sugar-ends content, expressed as glucose equivalent, was conducted as the indicator of the hydrolysis process. The representative reducing sugar content of the hydrosols is presented in Figure 2. The 0.1% cellulase incorporation in the composition of film-forming hydrosols caused a greater glucose equivalent than 0.05% of the enzyme during the same hydrolysis reaction time. After 3 and 20 h of hydrolysis, the difference in reducing sugar content between C0 and C0.1 was about 4 mM. The increase in reaction time increases the possibility of degrading CH/HPMC hydrosols by 0.05% cellulase, which was reflected in the increasing glucose concentration. The hydrolytic effect of cellulase on CH and HPMC was proven, and based on the results of the viscosity measurements and the 3,5-dinitrosalicylic acid (DNS) reducing sugar assay, we can conclude that 0.1% of tested enzyme and 3 h of hydrolysis are the best conditions to obtain low molecular weight chains of both polysaccharides. Shen et al. described commonly available and inexpensive enzymes, such as glycanases, cellulases, amylases, dextranases, hemicellulases, and pectinases with unexpected chitosanolytic and chitinolytic activities, capable of producing chitosan and chitin derivatives under ambient conditions [24].

### 2.3. DPPH (2,2-Diphenyl-1-picrylhydrazyl)

Statistical analysis of the ability to scavenge DPPH free radical and the ferric reducing/antioxidant power of hydrosols are presented in Table 1. The free radical scavenging activity method is widely used to determine the ability of substances to act as free radical scavengers or hydrogen donors. The DPPH radical is a stable, commercially available organic nitrogen radical which shows strong absorption band at 517 nm because of its single electron [25]. The absorption disappears when the single electron is quenched by the proton radical scavenger of the hydrogen-donating antioxidant and is transformed into a nonradical form [26]. The interactional influence of both factors was observed. The addition of cellulase and the prolonged reaction time to 20 h improved the scavenging effect of hydrosols (47.1% and 52.1% for C0.05*T*20 and C0.1*T*20, respectively) in comparison to hydrosol without enzyme (7.6%). A similar scavenging effect was noted for hydrosol treated with 0.1% cellulase for 3 h and hydrosol with lower concentration of enzyme, but for a longer time. DPPH free radical scavenging ability of hydrosols above 45.2%–47.1% may be obtained over a shorter time by the application of higher cellulase doses. The chitosan used in our study was from crab shells. Yen et al. [27] and Yen et al. [28] noted that crab chitosans are have good antioxidant properties—especially antioxidant activity, scavenging ability on hydroxyl radicals, and chelating ability on ferrous ions. Park et al. [29] previously reported higher a radical scavenging effect of hydroxyl, superoxide, alkyl, and DPPH radical of low molecular weight chitosan than that of a higher one. The antioxidant activity of chitooligomers and its derivatives is related to the amount and activity of the hydroxyl group at C6 and the amino group at C2 of the chitosan molecule [30]. The substitution of the chitosan functional group may reduce the amount of active amino and hydroxyl groups in the polymer chains, and may partly destroy the intermolecular and intramolecular hydrogen bonds [31].

### 2.4. Ferric Reducing Antioxidant Power (FRAP)

The mechanism of ferric reducing ion antioxidant power relies on the reduction of complex Fe^3+^-tripyridyltriazine to the Fe^2+^ form by an antioxidant in acidic conditions, and color changes are seen, with a maximum absorption at 593 nm [32]. Significant effects of cellulase addition and time of reaction on antioxidant power of the tested film-forming solutions were reported (Table 1). The enzyme dosage of 0.1% improved antioxidant power from 61.6 (without enzyme) to 87.9 µM Fe(II)/mL, from 81.6 to 266.2 µM Fe(II)/mL, and from 93.6 to 329.4 µM Fe(II)/mL after 0, 3, and 20 h of reaction, respectively. In addition, similar improvement was observed regarding lower concentrations of cellulose (−0.05%), and the antioxidant power was adequately lower (71.58, 161.30, and 275.6 µM Fe(II)/mL after 0, 3, and 20 h). It was noticed that samples of C0.1*T*20 statistically have the highest antioxidant power. A better antioxidant power result can be achieved by fractionation of hydrolyzed polysaccharides, as was proposed by Zimoch-Korzycka et al. [15]. They have proven that original chitosan used in the degradation process possess a good reducing capacity of 447.5 and 536.6 µM Fe(II)/mL, but was still worse than their insoluble fractions, ranging from 500 to 1350 µM Fe(II)/mL. The antioxidant properties could be impaired by the presence of HPMC in the hydrosol composition. Qin et al. indicated that with a lower degree of acetylation (DA) came a higher chitosan chelating activity [33]. The DA of chitosan used in our study was 15%–25%, which suggests good metal chelating ability. Metal ions present in food products may initiate the process of lipid peroxidation, causing flavor and taste deterioration, especially in raw meat [34].

### 2.5. Antimicrobial Activity

**Figure 3:**
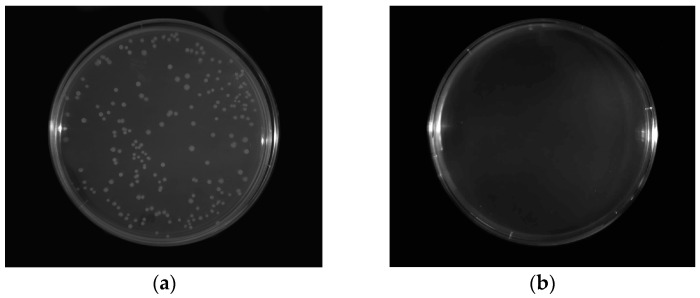
Antimicrobial effect: (**a**) growth of *Y. enterocolitica* (initial population); (**b**) inhibition of *Y. enterocolitica* growth with applied C0.1T20 hydrosol.

The antimicrobial effect of CH/HPMC hydrosols modified by cellulase is shown in Table 2. All tested bacterial log reductions were affected by cellulase addition and hydrolysis time of polysaccharide solutions. The total reduction of bacterial growth caused by the treatment of each hydrosol variant was observed against *Bacillus cereus*. The reduction was about 9.2 log cfu. The chitosan hydrolysates with viscosity varying from 5 to 10 cP inhibited the growth of *Bacillus* sp. isolated from fish meat paste at 50 ppm, which was reported by Cho et al. [35]. The C0.1*T*20 hydrosol was characterized with a viscosity of 13.0 cP after 20 h of hydrolysis. Our result was as effective as described by Zimoch-Korzycka and Jarmoluk [36]. They have found that 10^4^ log cfu/mL of *B. cereus* was completely inactivated as a result of the simultaneous effect of chitosan, lysozyme, colloidal silver, and hydroxypropyl methylcellulose. Components of the tested hydrosols were less efficient against the growth of other Gram-positive bacteria; *Staphylococcus aureus* induced 100% reduction in C0.1*T*3, C0.05*T*20, and C0.1*T*20. Xiao et al. [37] found that chitosan/arginine with a higher concentration of positive charges possesses decreasing antibacterial activity against *S. aureus*. They concluded that positively-charged free –NH^3+^ and/or the guanidine groups of chitosan or chitosan/arginine may bind strongly to the components of the cell wall, leading to pore formation in the cell walls, causing the leakage of cell constituents, resulting in cell death. When the concentration of chitosan and chitosan/arginine is too low, they are not able to destroy bacterial cells by disrupting–distorting, and chitosan and chitosan/arginine become nourishment for bacteria. This suggests that better antimicrobial activity of modified chitosan does not depend on the higher cationic charge in its molecule [37]. This also explains our reduced inhibition result against *S. aureus* compared to other tested bacteria. The bactericidal efficiency of chitosan is multifaceted and is connected to different factors, which must be considered in the evaluation of its action. These parameters were categorized into four classes by Kong et al. [38]: (1) microbial factors—the microbial species and the cell age; (2) intrinsic factors of chitosan molecules—the positive charge density, the protonation level of the amine group, the molecular weight, the concentration, the hydrophilic/hydrophobic characteristics, and the chelating capacity; (3) physical state of chitosan—soluble and solid; and (4) environmental factors—the ionic strength, the pH, the temperature, and the contact time between chitosan and bacterial cells. Gram-negative bacteria were sensitive to the action of cellulase and the time of its reaction. A simultaneous total reduction effect of these factors on *P. fluorescens* was noted for each variant to which cellulase was added. *Y. enterocolitica* growth was completely reduced only in the cases of C0.1*T*3, C0.05*T*20, and C0.1*T*20 (Figure 3a,b). The mode of antibacterial activity is a process that differs between Gram-positive and Gram-negative bacteria, due to different cell surface structures. The anionic phosphate and carboxyl groups present in lipid components and the inner core of the lipopolysaccharide stabilize the lipopolysaccharide layer of the Gram-negative outer membrane through electrostatic interactions with divalent cations. A chelating agent may remove this cation and release lipopolysaccharide, destabilizing the outer membrane and causing death of the bacteria [39].

## 3. Materials and Methods

### 3.1. Materials

Low molecular weight chitosan (decetylation degree (DD) = 75%–85%), DL lactic acid (85% syrup), and glycerol 99% were obtained from Sigma Aldrich, Poznań, Poland. Hydroxypropyl methylcellulose—HPMC (Methocel) was purchased from Dow Chemical Co, Midland, MI, USA. Cellulase (CP CONC) with an activity 120 U/mg and side activity (typical) of 30 U/mg of β-glucanase was produced by the fermentation of non-GMO *Trichoderma longibrachiatum* (formerly *Trichoderma reesei*) and obtained from Dyadic (Jupiter, FL, USA).

### 3.2. Hydrosols Preparation

Cellulase stock solution was prepared by dissolving in bidistilled water and centrifugation (5000× *g*). The HPMC was dissolved in bidistilled water, and the CH was solubilized in 2% (*v*/*v*) aqueous lactic acid solutions at 1%. HPMC and CH were blended with 25% (*w*/*w*) of glycerol (of dry weight of the used polymers) and cellulase (at three different levels: 0%, 0.05%, and 0.1%) in different ratio (*w*/*w*), as shown in Table 3. Hydrosols were boiled for 30 min to inactivate the enzyme after 3 and 20 h of hydrolysis at 24 °C. The time of hydrolysis was determined by measuring the viscosity and reducing sugars of hydrosols.

### 3.3. Viscosity Determination

Viscosity of film-forming hydrosols was performed in Haake RS 6000 rotational viscometer (Thermo Scientific, Karlsruhe, Germany). The measurement was made at a constant temperature (24 °C) using a system of coaxial cylinders with conical rotor Z20 DIN. Sample (8.2 mL) was applied to the cylinder and analyzed with a constant shear rate of 10 s^−1^ for 22 h.

### 3.4. Quantitive Determination of Reducing Sugar-Ends

The determination of reducing sugar-ends was performed using 3,5-dinitrosalicylic acid (DNS), according to Miller [40]. DNS reagent solution contained 3,5-dinitrosalicylic acid (1 g), 2 M NaOH (20 mL), and potassium sodium tartrate (30 g) in distilled water (100 mL). Hydrosols were mixed with the reaction reagent of DNS in a ratio of 1:1 (*v*/*v*) and were heated for 8 min. After cooling and centrifugation, the absorbance was measured at a wavelength of 540 nm. The amount of reducing sugars was read from standard calibration curve of d-glucose.

### 3.5. Free Radical Scavenging Activity

Free radical scavenging activity of the hydrosols was determined by the method of Chen et al. [41]. First, 1 mL of 0.1 mM DPPH (2,2-diphenyl-1-picrylhydrazyl)-methanol solution was incubated with 1 mL hydrosol. The reaction mixture was shaken well and incubated for 30 min at ambient temperature. The reduction of the DPPH free radical was measured by reading the absorbance at 517 nm. Control sample was DPPH-methanol solution. The antioxidant activity was calculated from the standard curve and expressed in µM Trolox/mL needed to neutralize 0.15 mM solution of DPPH free radicals.

### 3.6. Ferric Reducing Ion Antioxidant Power

The antioxidant power of hydrosols was determined by the method described by Benzie and Strain [42]. Hydrosol (100 µL) was mixed with FRAP reagent (3 mL), and absorbance was read at 593 nm. Standard calibration solutions were prepared with ferrous sulphate (0–1 mM).

### 3.7. Antimicrobial Activity

The method was described by Song et al. [43]. The following microorganisms were used in the test: *Pseudomonas fluorescens* PCM2123 (Polish Collection of Microorganism), *Yersinia enterocolitica* PCM1889, *Bacillus cereus* PCM2003, *Staphylococcus aureus* PCM1932. The bacterial cultures were grown on a nutrient agar slant and kept at 4 °C. Microorganisms were taken from the slant and transferred to nutrition broth (BTL sp. zo.o., Łódź, Poland) for propagation for 18 h at 37 °C. The bacterial suspension was standardized by transferring 1 mL of the culture to a fresh medium to a density of 1 McFarland scale. The absorbance was measured at a wavelength of λ = 550 nm to determine cell density and obtain 10^6^ cfu/mL of bacterial culture concentration. Bacterial cell suspension (0.1 mL) was introduced into 1 mL of hydrosol and incubated at 37 °C for 30 min. Then, the samples were applied in an amount of 0.1 mL of the suspension on a plate of solidified agar. The plates were incubated at 37 °C for 24 h. After this time, the grown colonies were counted. The results are presented as log reduction and were calculated by following equation:

$$Log\;reduction=\;log_{10}\left(N_{0}\right)-log_{10}\left(N\right)$$

where *N*_0_ is the number of viable microorganisms before treatment (initial population) and *N* is the number of viable microorganisms after treatment by hydrosols.

### 3.8. Statistical Analysis

Data were analyzed by two-way Anova using Statistica 9. Mean values and standard errors (SE) of the mean were reported. The differences between the means of DPPH and FRAP results were established with Duncan Test with 5% significance. All experiments were performed in triplicate.

## 4. Conclusions

The enzymatic activity of cellulase was confirmed in viscosity test and DNS assay. The viscosity value of polysaccharides solutions treated by enzyme in two tested concentrations varied slightly. The differences were observed in the reducing sugars results. A reduced viscosity allows the production of thinner films or coatings. The radical scavenging ability of film-forming hydrosols was improved by enzymatic hydrolysis, and shows their potential to be widely used in the food industry. The effectiveness of the selected hydrosols against the tested microorganism was 100%. In conclusion, *B. cereus* is sensitive to the action of chitosan, which was seen in the total reduction of each tested variant. Maintaining food quality and safety could be possible by the use of natural substances, such as chitosan or chitooligomers, which are able to reduce microbial growth. The use of these substances also improves antioxidant properties, which may be helpful to limit or prevent oxidation processes, such as color deterioration of meat or lipid oxidation. The results of the antimicrobial and antioxidant activities of the tested hydrosols are very promising, and therefore may be applied in other food products, drugs, or tissue engineering.

## Figures and Tables

**Table 1:** Effects of cellulase (C) addition (%) and time of reaction (*T*, h) on antioxidant properties of film-forming hydrosols. DPPH: 2,2-diphenyl-1-picrylhydrazyl; FRAP: ferric reducing antioxidant power.

Variants Coding	Scavenging Effect of DPPH (%)	FRAP (µM Fe(II)/mL)
C0*T*0	7.6 ± 0.73 ^a,b^	61.6 ± 7.08 ^a^
C0.05*T*0	9.2 ± 0.57 ^a,b^	71.6 ± 4.74 ^a,b^
C0.1*T*0	11.5 ± 2.16 ^b^	87.9 ± 6.16 ^b,c^
C0*T*3	6.8 ± 1.57 ^a^	81.6 ± 2.97 ^a,b,c^
C0.05*T*3	39.5 ± 2.13 ^c^	161.3 ± 7.70 ^d^
C0.1*T*3	45.2 ± 0.14 ^d^	266.2 ± 13.29 ^e^
C0*T*20	5.9 ± 0.49 ^a^	93.6 ± 3.76 ^c^
C0.05*T*20	47.1 ± 2.46 ^d^	275.6 ± 15.93 ^e^
C0.1*T*20	52.1 ± 5.50 ^e^	329.4 ± 25.07 ^f^

Results are expressed as mean ± standard deviation (SD). Values with different letters ^(a–f)^ within the same column differ significantly (*p* < 0.05).

**Table 2:** Effects of cellulase addition (C, %) and reaction time (*T*, h) on the antimicrobial properties of film-forming hydrosols.

Variants Coding	Log Reduction			
	*P. fluorescens*	*Y. enterocolitica*	*B. cereus*	*S. aureus*
C0*T*0	2.2 ± 0.26	0.3 ± 0.13	9.2 ± 0.06	0.9 ± 0.13
C0.05*T*0	9.5 ± 0.05	0.3 ± 0.06	9.2 ± 0.06	2.0 ± 0.23
C0.1*T*0	9.5 ± 0.05	0.5 ± 0.07	9.2 ± 0.06	2.2 ± 0.06
C0*T*3	4.7 ± 4.12	0.3 ± 0.11	9.2 ± 0.06	0.9 ± 0.27
C0.05*T*3	9.5 ± 0.05	1.5 ± 0.32	9.2 ± 0.06	1.7 ± 0.48
C0.1*T*3	9.5 ± 0.05	9.3 ± 0.07	9.2 ± 0.06	9.2 ± 0.06
C0*T*20	2.3 ± 0.23	0.7 ± 0.26	9.2 ± 0.06	1.4 ± 0.10
C0.05*T*20	9.5 ± 0.05	9.3 ± 0.07	9.2 ± 0.06	9.2 ± 0.06
C0.1*T*20	9.5 ± 0.05	9.3 ± 0.07	9.2 ± 0.06	9.2 ± 0.06

Results are expressed as mean ± standard deviation (SD).

**Table 3:** Experimental design.

Variants Coding	Variation Factors		Constant Factors			
	Cellulase (%)	Time (h)	Chitosan (%)	HPMC (%)	Glycerol (%)	Lactic Acid (%)
C0*T*0	0	0	1	1	25	0.25
C0.05*T*0	0.05					
C0.1*T*0	0.1					
C0*T*3	0	3				
C0.05*T*3	0.05					
C0.1*T*3	0.1					
C0*T*20	0	20				
C0.05*T*20	0.05					
C0.1*T*20	0.1					
